# Halted medical education and medical residents’ training in Korea, journal metrics, and appreciation to reviewers and volunteers

**DOI:** 10.3352/jeehp.2025.22.1

**Published:** 2025-01-13

**Authors:** Sun Huh

**Affiliations:** Institute of Medical Education, Hallym University College of Medicine, Chuncheon, Korea; The Catholic University of Korea, Korea

## A break in medical education and medical resident training in Korea

On the night of December 3, 2024, just after 10 PM, television was abruptly interrupted by a Korean presidential emergency briefing announcing martial law. The ensuing English and Korean versions of the proclamation are available in Supplement 1. Out of 6 items in the martial law proclamation, the 5th was especially alarming:

All medical professionals, including medical residents who are currently striking or have left their medical posts, must return to their duties within 48 hours and perform their roles diligently. Failure to comply will result in punishment under martial law.

Upon reading this, I was disturbed because as of December 3, 2024, there were no striking medical professionals; nonetheless, the proclamation threatened punishment under martial law if they did not “return to work” within 48 hours. Fortunately, the martial law was rejected by the National Assembly of Korea on December 4, 2024, and no further action was taken.

*Why was this baseless item included in the martial law proclamation?* On February 20, 2024, most medical residents in Korea began to resign from their training hospitals after the Ministry of Health and Welfare announced an increase in the medical school admission quota by 2,000—from 3,058 to 5,058—starting in 2025 [1]. In the absence of dialogue with the medical profession, this abrupt increase was imposed with neither consensus nor tangible support. Some residents found new jobs or voluntarily worked in various facilities after resigning from training hospitals. Moreover, the majority of medical students submitted formal leave-of-absence requests to their universities [2]. The Ministry of Health and Welfare insisted that the increase was justified based on 3 reports [3-5]; however, the authors of those reports contended that their data could not serve as the basis for such an abrupt increase [1].

Last semester, I taught only one student in my parasitology class, since all the other students were on leave of absence. This phenomenon impacted the number of applicants for the Korean Medical Licensing Examination (KMLE). In the 2024 administration of the clinical practice examination, 347 candidates participated, of whom 266 passed, yielding a 76.7% success rate. For the written examination scheduled in January 2025, only 310 individuals registered—about 1/10 the typical enrollment figure [6]. It is saddening for educators not to encounter the students or residents they would normally teach.

I hope the current issues regarding the medical school admission quota will be smoothly resolved after dialogue between the government officers and the medical profession based on scientific evidence. Moreover, the government must issue a sincere apology for the proclamation that threatened medical residents with oppressive rhetoric and pledge to prevent any recurrence. Additionally, a strategy is urgently required to address and heal the fear these individuals have endured.

## Highlights in the 2024 issue

In 2024, the *Journal of Educational Evaluation for Health Professions* (*JEEHP*) published 41 articles, including 5 editorial materials. Similar to 2023 [7], many studies focused on the performances of generative artificial intelligence (AI). Topics included AI-based medical imaging evaluation, performance on simulated European Board of Interventional Radiology examinations, standardized urology knowledge, and the Japanese National License Examination for Pharmacists in 2022. An additional study described the development of continuing professional development plans for graduate radiographers using ChatGPT-4o [8]. Despite adhering to broad regulatory standards, ChatGPT-4o exhibited limitations in addressing individualized and context-specific needs.

Kim et al. [9] contributed a valuable review article on the legality and appropriateness of keeping Korean Medical Licensing Examination items confidential. They concluded that exam items qualify as non-public information under the Korea Official Information Disclosure Act, allowing the Korea Health Personnel Licensing Examination Institute to maintain confidentiality to ensure fairness and efficiency.

Another noteworthy contribution addresses the Dr. LEE Jong-wook Fellowship Program implemented by the Korea Foundation for International Healthcare in memory of the late WHO Secretary-General. Since 2007, the program has guided 1,504 practitioners from 30 nations, enabling them to gain advanced Korean medical knowledge. Oh and Yoon [10] developed a new performance evaluation indicator for assessing this fellowship’s long-term educational impacts through Delphi surveys.

## What will be the next topics?

It has been 20 years since I took on the role of editorship in 2005. The journal has been promoted internationally from the standpoint of diverse authors’ countries, indexing in international literature databases, and citation frequencies in the indexing databases. Furthermore, appropriate study designs and the corresponding reporting guidelines have been strictly required since 2020 to maintain scientific soundness. The primary concern in accepting manuscripts is their practical usefulness in health professions education. If educators and students can benefit in any way from journal articles, I and all editorial board members and staff will be happy.

The primary scope of the journal is the licensing examinations for health professions throughout the world. However, it is challenging to find those topics in the literature databases. This may originate from the difficulty in accessing the results of licensing examinations in each country. Therefore, JEEHP is unique in this field. The second concern is the adoption of measurement theory within the educational framework of health professions. JEEHP has frequently recruited manuscripts on this topic. Many other articles are for measurement tools development, program evaluation, and introduction of new technologies. The recent most striking topics have been applications of the metaverse and generative artificial intelligence.

What will be the next hot and useful topics for the future of health professions education? With technical developments, virtual reality through smart glasses will become more user-friendly. Deep learning and large language models have already emerged as common subjects in health professions education. Students should understand and use those tools without difficulties. Furthermore, deep learning and large language models should be utilized throughout the learning process.

The timeline for the availability of quantum computing remains uncertain. However, once it becomes accessible, the integration of quantum computing with deep learning is expected to significantly transform various fields, including drug development [11], radiological diagnosis, tailored chemotherapy, and genomic analysis [12]. To prepare for the future medical environment, should we consider introducing the concept and use of quantum computing in health professions curricula? Although it remains a dream to have quantum computing at our desks, scientific development can soon break through the present limitations of a quantum computing environment. The editorial board welcomes submissions of manuscripts on these new topics and encourages contributions on any other special topics relevant to the journal.

## Journal metrics and statistics of the 2024 issue

After receiving the first official Journal Impact Factor (JIF) of 4.4 in June 2023, JEEHP’s 2023 JIF reached 9.3 in June 2024 [7], the highest ranking in the scientific education category. The Citescore 2024 by Elsevier was 15.5 on December 31, 2024, with most citations concentrated in articles published from 2021 to 2023 (Table 1).

According to these citation results, it is an urgent priority for the editor to recruit highly citable research articles, even though review articles generally receive more citations in most journals.

**Fig. 1.**
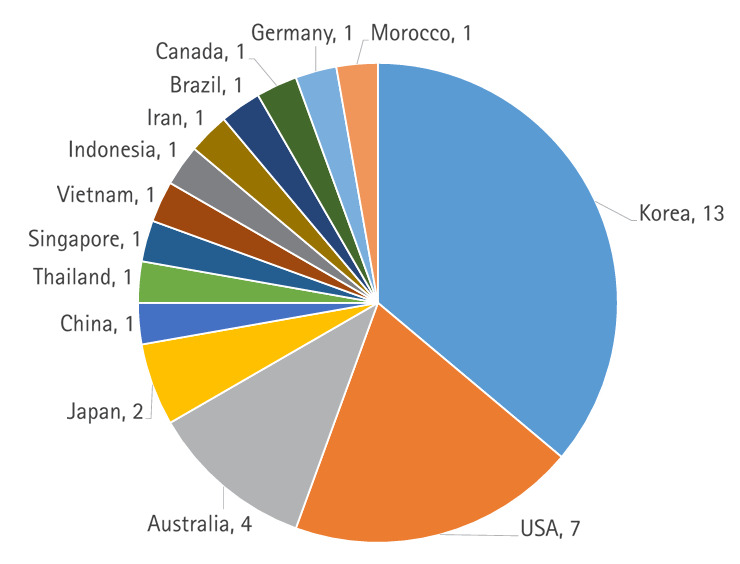
Authors’ countries of 36 articles published in the *Journal of Educational Evaluation for Health Professions* in 2024. Five editorial materials were excluded.

Fig. 1 shows the authors’ countries of 36 articles in the 2024 issue, from which 3 editorials, 1 erratum, and 1 corrigendum were excluded. The proportion of articles from Asian countries was 58.3%, which aligns with the journal’s regional scope.

Journal statistics for 2024 are presented in Table 2. The data for 2024 were somewhat different from the 2023 data [7]. The number of unsolicited manuscripts increased from 261 in 2023 to 337. The acceptance rate of unsolicited manuscripts decreased from 11.9% in 2023 to 8.8%. The editorial office has done its best to minimize the rejection rate of peer-reviewed manuscripts. However, the acceptance rate of peer-reviewed manuscripts decreased from 80.6% in 2023 to 74.4%. Those changes in journal metrics were inevitable since the publication budget (space) is limited.

## Appreciation to reviewers and volunteers

In 2024, 100 reviewers were invited from 24 countries, listed below. Without their invaluable time and expertise, this journal could not maintain its quality. We extend our deepest gratitude for their support:

**Australia:** Matthew Rose (Australian Resuscitation Council), Amy Grayton Carroll, Kylie Kendrick (Deakin University), Gomes Yolanda (Flinders Medical Centre), Elias Biris (Royal Adelaide Hospital), Taylor Stephanie (The Queen Elizabeth Hospital)
**Canada:** Arora Rohit (IBM Canada)
**Chile:** Rosel Margarita (Universidad Andres Bello), Antonio Cespedes (Private dental practice affiliated with the University of the Andes)
**China:** Libin Huang (Sun Yat-Sen University), Jianhua Feng (University of Chinese Academy of Sciences)
**Czech:** Vera Spatenkova (Technical University of Liberec)
**Germany:** Jonathan Kotltors (University Hospital Cologne), Manuel Florian Struck (University Hospital Leipzig)
**India:** Ayeesha Juhi (All India Institute of Medical Sciences)
**Indonesia:** Rahma Tsania Zhuhra (Universitas Andalas), Wahid Mardiastuti (Universitas Indonesia)
**Iran:** Khalil Momeni (Ilam University), Heresh Moridi (Kurdistan University of Medical Science)
**Italy:** Ferrara Vincenza (Sapienza University of Rome)
**Japan:** Kuribara Tomoki (Sapporo City University)
**Korea:** Sukhyang Lee (Ajou University), Jonggi Choi (Asan Medical Center), Sung Woo Hwang (Baekseok Culture University), Young Youn Cho, Kwisoon Choe (Chung-Ang University), Sang Woong Park (Eulji University), Kyung-Nyun Kim (Gangneung-Wonju National University), Dong Gi Seo, Geum-Hee Jeong, Jeongwook Choi, Yera Hur, Younjae Oh (Hallym University), Jong Geun Yun (Honam University), Yeonjoo Seo (Inflolumi), Yongsang Lee (Inha University), Bo Young Yoon (Inje Universtiy Ilsan Paik Hospital), Byoung-Gun Park (Jeonbuk National University), Daegyu Sung (Kangwon National University), Hyun Sook Yi (Konkuk University), YoungMin Park (Korea Health Personnel Licensing Examination Institute), Yanghyun Chun (Kyun Hee University), Jungkyu Park (Kyungpook National University), Yoonjung Lee (Seoul National University College of Medicine), A Ra Cho, Soojung Kim, Sun Kim, Wha Sun Kang (The Catholic University of Korea), Yoon-Seon Lee (University of Ulsan), Chun Il Park, Han Joe Kim, Hyeyeon Park, Sue Kim, Yeunsoo Yang, Hanna Jung, Byung Il Yeh, Kang Huyn Lee (Yonsei University)
**Morocco:** Qaisar Rabia (Higher Institute of Nursing and Health Techniques), Houmam Idriss (Ibn Zohr University), Driss Ait Ali (Sidi Mohammed Ben Abdellah University)
**Netherlands:** Lukas Stalpers (Amsterdam University Medical Center)
**New Zealand:** Marcus Henning (University of Auckland)
**Pakistan:** Talat Hajra (Rahbar Medical College), Ali Fizzah (University of Lahore)
**Singapore:** Jason Lee (Duke NUS Medical School), Derrick Lian, Dujeepa D. Samarasekera, Shuh Shing Lee, Chng Hui Ting (National University of Singapore)
**Switzerland:** Sylvain Boloré (HES-SO University of Applied Sciences and Arts)
**Taiwan:** Yu-Chun Chiu (National Taiwan University), Ying-Ying Yang (Taipei Veterans General Hospital)
**Thailand:** Sombat Tayraukham (Chiang Mai University), Sittichai Thongworn (Mahidol University), Chankong Warangkana (Sukhothai Thammathirat Open University)
**Turkey:** Sezer Hale (Bakırcay University), Dincer Beste (Aydin Adnan Menderes University)
**UAE:** Adnan Agha, Munawar Farooq (UAE University)
**USA:** Damian Keter (Cleveland VA Medical Center), Wimsatt Leslie (Des Moines University), Brian Alverson (Jefferson University), Jay Fuletra (Main Line Health), Peggy Hsieh (McGovern Medical School), Nicole Hodges, Paul Craig (Methodist University), Scott Geiger (Mt. Nittany Medical Center), Claudia Der-Martirosian (Oregon Community Health Information Network), Wang Yi (Purdue University), Wu Tong (Riverside Insights), Rassbach Caroline (Stanford Healthcare), Taylor Stephanie (The Queen Elizabeth Hospital), Farhad Hagigi (University of California, Los Angeles), DeShetler Lori (University of Toledo), Myunghee Jun (University of Wisconsin-Green Bay), Rachel Moquin (Washington University)
**Vietnam:** Nguyen Thi Nhan (University of Medicine and Pharmacy at Ho Chi Minh City)
**Unidentified:** Adham El Sahfie, Ismail Yahya

If any reviewers in 2024 are not listed here, it is the editor’s responsibility. Please contact us to notify us of any omissions.

## Figures and Tables

**Table 1.** Highly cited articles in the *Journal of Educational Evaluation for Health Professions* published from 2021 to 2023 [cited 2024 Dec 31]

No.	Title	Year	Publication type	Citation frequency	
				WOS	Scopus
1	Sample size determination and power analysis using the G*Power software	2021	Review	829	875
2	Educational applications of metaverse: possibilities and limitations	2021	Review	319	572
3	Are ChatGPT’s knowledge and interpretation ability comparable to those of medical students in Korea for taking a parasitology examination?: a descriptive study	2023	Brief report	176	199
4	E-learning in health professions education during the COVID-19 pandemic: a systematic review	2021	Review	86	108
5	Training in lung cancer surgery through the metaverse, including extended reality, in the smart operating room of Seoul National University Bundang Hospital, Korea	2021	Editorial	70	101
6	Can an artificial intelligence chatbot be the author of a scholarly article?	2023	Review	54	69
7	Application of computer-based testing in the Korean Medical Licensing Examination, the emergence of the metaverse in medical education, journal metrics and statistics, and appreciation to reviewers and volunteers	2022	Editorial	31	35

WOS, Web of Science.

**Table 2.** Journal statistics of manuscripts submitted to the *Journal of Educational Evaluation for Health Professions* from January 1 to December 31, 2024

	No.	Content
Manuscripts submitted	345	
No. of commissioned manuscripts and editorial materials	8	Editorials, 3; reviews, 1; software, 1; correspondence, 1; erratum, 1; corrigendum, 1
No. of unsolicited manuscripts	337	
Manuscripts rejected without peer review	289	Unsuitable, 280; withdrawal, 1; other reasons, 6; rejected before review, 2
Manuscripts peer-reviewed out of 337 submitted manuscripts	48	Published, 29; rejected, 10; under review, revision or editing, 8; duplicate and excluded, 1
No. of publications out of 39 peer-review finished unsolicited manuscripts	29	Acceptance rate of only peer-reviewed manuscripts: 29/(48–9)=29/39=74.4%
Acceptance rate overall (%)	11.0	37/(345–9)=37/336=0.110
Acceptance rate of unsolicited manuscripts (%)	8.8	29/(337–9)=29/328=0.088
Median time from submission to the first decision (day)	14	
Median time from submission to publication (day)	35	
Median time from acceptance to publication (day)	3	

## Supplementary materials

Supplementary files are available from Harvard Dataverse: https://doi.org/10.7910/DVN/XFZX8U

Supplement 1. Martial law proclamation by the Martial Law Commander, Army General Park An-Soo (Proclamation No. 1).
