# Supplementary material for: Halted medical education and medical residents’ training in Korea, journal metrics, and appreciation to reviewers and volunteers
Source: J Educ Eval Health Prof. 2025 Jan 13;22:1. doi: 10.3352/jeehp.2025.22.1 (PMC11880820; doi:10.3352/jeehp.2025.22.1)
Supplement: Supplementary file 1 — Supplement 1. Martial law proclamation by the Martial Law Commander, Army General Park An-Soo (Proclamation No. 1). [file jeehp-22-01-suppl1.pdf]

Supplement 1. Martial law proclamation by the Martial Law Commander, Army General Park An-Soo (Proclamation No. 1)

<English version>

Martial law proclamation by the Martial Law Commander, Army General Park An-Soo (Proclamation No. 1)

To protect liberal democracy from the threats of anti-state forces conspiring to overthrow the Republic of Korea and to ensure the safety of the people, the following measures are hereby proclaimed nationwide effective December 3, 2024, at 11:00 PM.

1. All political activities, including those of the National Assembly, local assemblies, political parties, political gatherings, assemblies, and protests, are prohibited.
2. Any actions that deny or attempt to overthrow the liberal democratic system, as well as the dissemination of fake news, manipulation of public opinion, and false propaganda, are forbidden.
3. All media and publications will be controlled by the martial law authorities.
4. Strikes, work stoppages, and assemblies that incite social chaos are prohibited.
5. All medical professionals, including medical residents who are currently striking or have left their medical posts, must return to their duties within 48 hours and perform their roles diligently. Failure to comply will result in punishment under martial law.
6. Honest and law-abiding citizens, excluding anti-state elements and those seeking to overthrow the system, will be accommodated to minimize disruptions to their daily lives.

Proclamation issued on December 3, 2024 (Tuesday) by the Martial Law Commander, Army General Park An-Soo

<Korean version>

계엄사령부 포고령(제1호)

자유대한민국 내부에 암약하고 있는 반국가세력의 대한민국 체재전복 위협으로부터 자유민주주의를 수호하고, 국민의 안전을 지키기 위해 2024년 12월 3일 23:00 부로 대한민국 전역에 다음 사항을 포고합니다.

1. 국회와 지방의회, 정당의 활동과 정치적 결사, 집회, 시위 등 일체의 정치활동을

금한다.

2. 자유민주주의 체재를 부정하거나, 전복을 기도하는 일체의 행위를 금하고, 가짜뉴스, 여론조작, 허위선동을 금한다.

3. 모든 언론과 출판은 계엄사의 통제를 받는다.

4. 사회혼란을 조장하는 파업, 태업, 집회 행위를 금한다.

5. 전공의를 비롯하여 파업 중이거나 의료현장을 이탈한 모든 의료인은 48시간 내 본업에 복귀하여 충실히 근무하고 위반시는 계엄법에 의하여 처단한다.

6. 반국가 세력 등 체제 전복 세력을 제외한 선량한 일반 국민들은 일상생활에 불편을 최소화할 수 있도록 조치한다.

2014년 12월 3일 (화) 계엄사령관 육군대장 박안수
